# Two Cases of Ectopic Hamartomatous Thymoma Masquerading as Sarcoma

**DOI:** 10.1155/2017/1672919

**Published:** 2017-01-10

**Authors:** Takahito Kondo, Yukiko Sato, Hiroko Tanaka, Toru Sasaki, Kazuyoshi Kawabata, Hiroki Mitani, Hiroyuki Yonekawa, Hirofumi Fukushima, Wataru Shimbashi

**Affiliations:** ^1^Department of Head and Neck Oncology, Cancer Institute Hospital of Japanese Foundation for Cancer Research, 3-8-31 Ariake, Koutou-ku, Tokyo 135-8550, Japan; ^2^Department of Pathology, Cancer Institute Hospital of Japanese Foundation for Cancer Research, 3-8-31 Ariake, Koutou-ku, Tokyo 135-8550, Japan; ^3^Department of Diagnostic Imaging, Cancer Institute Hospital of Japanese Foundation for Cancer Research, 3-8-31 Ariake, Koutou-ku, Tokyo 135-8550, Japan

## Abstract

Ectopic hamartomatous thymoma (EHT) is an extremely rare benign tumor. EHTs are difficult to differentiate from sarcomas, especially synovial sarcomas. We encountered two cases of EHT that were referred from other hospitals because sarcoma was suspected. In these cases, fusion gene detection via polymerase chain reaction or fluorescence in situ hybridization was useful for differentiating EHT from synovial sarcoma. EHT requires accurate diagnosis before surgery to avoid excessive treatment. Both tumor location and the presence of fat inside the tumor are important imaging findings for EHT, and confirmation of spindle cells, epithelial cells, and mature adipose cells in the tumor is an important pathological finding. It is important to exclude synovial sarcoma from the differential diagnosis via fusion gene analysis.

## 1. Introduction

Ectopic hamartomatous thymoma (EHT) is a benign tumor that occurs with extreme rarity in the lower neck. Although EHT disease is referred to as a thymoma, there is no evidence of thymic origin or differentiation [1]. Because EHT is an extremely rare tumor, it is often difficult to diagnose. Additionally, EHT is difficult to differentiate from synovial sarcoma (SS) or other malignant tumors. If EHT is diagnosed incorrectly, treatment leads to unnecessary resection.

Here, we report two cases of EHT in whom the differentiation of EHT from SS was assisted by fusion gene detection using polymerase chain reaction (PCR) or fluorescence in situ hybridization (FISH), as genetic diagnostic techniques [2].

## 2. Case Presentation

### 2.1. Case 1

**Figure 1:**
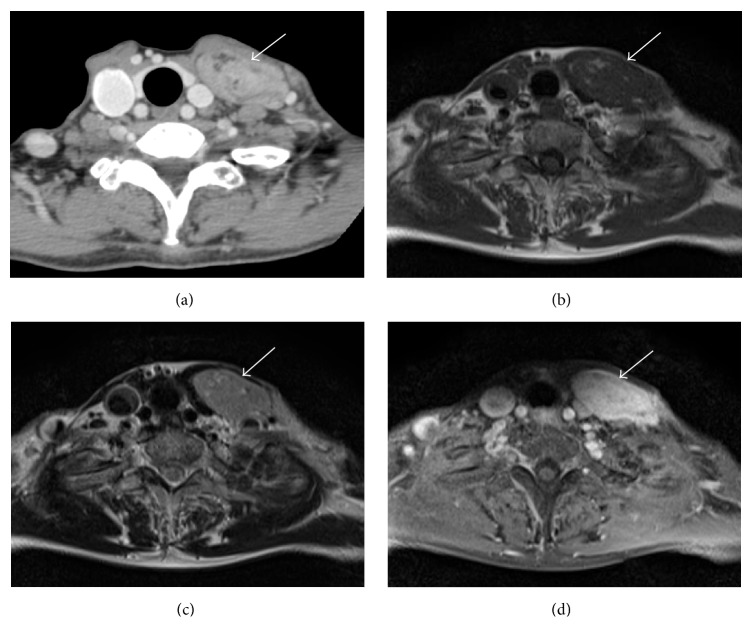
Preoperative imaging findings for Case 1. (a) Contrast-enhanced computed tomography image. T1-weighted axial (b), T2-weighted axial (c), and fat-suppressed gadolinium-enhanced T1-weighted axial (d) magnetic resonance images. Computed tomography and magnetic resonance images show a soft tissue tumor with adipose tissue between the sternocleidomastoid muscle and the anterior strap muscle.

A 60-year-old woman had been aware of a mass in her left lower neck for 5 months. She underwent an open biopsy of the left neck by a previous physician. The findings of the histopathological examination indicated spindle cell sarcoma. On physical examination, an extremely soft mass was palpated in the left lower neck. Computed tomography (CT) scans (Figure 1(a)) revealed a 60 × 26 × 42 mm well-marginated soft tissue mass in the left lower anterior neck. The sternocleidomastoid muscle was markedly displaced anteriorly. The mass presented with heterogeneous enhancement and low-density areas, suggesting the presence of intralesional fat. On magnetic resonance imaging (MRI), the tumor exhibited soft tissue intensity with scattered high intensity that was suggestive of fat in T1-weighted images (WI) (Figure 1(b)) and T2WI (Figure 1(c)). In fat-suppressed gadolinium-enhanced T1WI (Figure 1(d)), the mass showed marked enhancement, and high-intensity areas were suppressed. On ^18^fluorodeoxyglucose positron emission tomography/CT (FDG-PET/CT) imaging, the maximum standard uptake volume in the tumor was 2.1. Histopathological examination of the previous open biopsy revealed spindle cells with epithelioid differentiation. On immunohistochemical examination, the spindle cells and epithelial cells were cytokeratin (CK) AE1/3 (+). The spindle cells were alpha-smooth muscle actin (*α*-SMA) (+), S100 (−), desmin (−), Bcl2 (+), and terminal deoxynucleotidyl transferase (TdT) (−). SS and spindle cell carcinoma were suspected.

The tumor was resected from the left neck under general anesthesia. The scar created by the previous open biopsy was also resected. The sternocleidomastoid muscle around the tumor was also resected. Resection progressed along the deep cervical fascia with attaching adipose tissue surrounding the tumor. A phrenic nerve running to the deep portions of the tumor was resected. The tumor was detached from the surrounding tissue. Intraoperative frozen section examination results indicated the presence of a spindle cell tumor.

**Figure 2:**
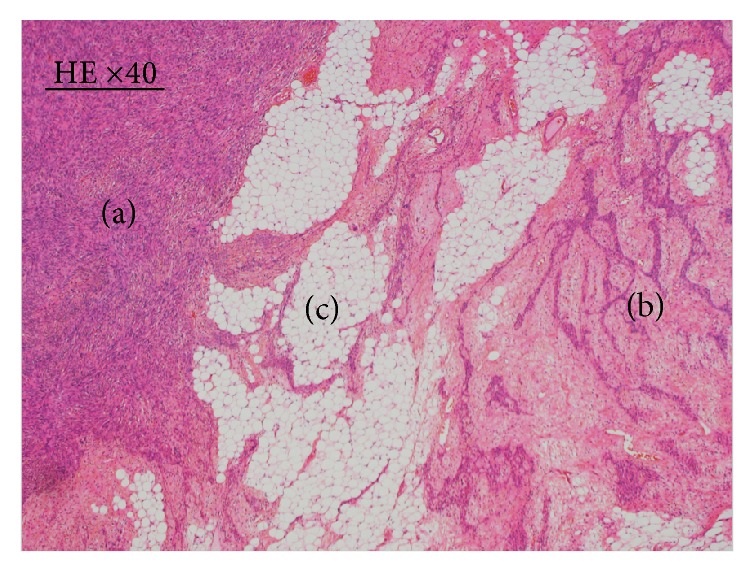
Hematoxylin and eosin (HE) staining findings in Case 1 (×40). (a) Plump to thin spindle cells with a low degree of cellular atypia can be observed. (b) Epithelial cells form anastomosing cords. (c) Intermingled adipose cells are present in the tumor.

We used frozen samples to assess SYT-SSX fusion gene expression by PCR. cDNA was prepared from total RNA extracted from the excised specimen. We used the SYT51 (5′-cagggaccacctccacaacag-3′) and SSX124-31 (5′-cctctgctggcttcttgggc-3′) primers, which recognize SSX1/2/4, to perform the 35-cycle PCR. The SYT-SSX fusion gene was not expressed, and thus SS was eliminated from the differential diagnosis. On histopathological examination of the resected tumor, the tumor consisted of plump to thin spindle cells growing in a bundle shape (Figure 2(a)), epithelial cells that formed anastomosing cords (Figure 2(b)), and epithelial islands with solid or microcystic extension areas. In addition, mature adipose cells (Figure 2(c)) and lymphocytes were observed between spindle cells and epithelial cells. Upon evaluation of the overall lesion, we diagnosed the tumor as EHT. The patient has had no recurrence for 56 months after surgery. The patient provided written informed consent for all medical procedures and for the publication of this case report.

### 2.2. Case 2

**Figure 3:**
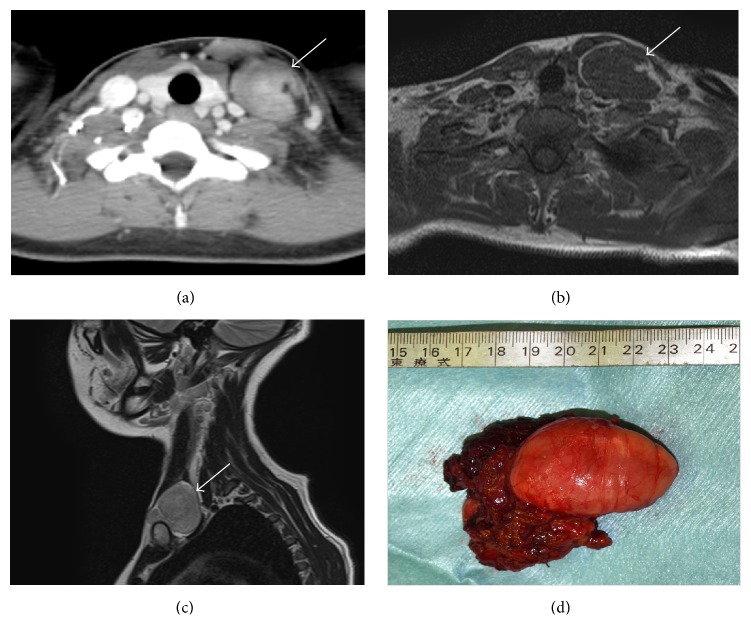
Preoperative imaging findings for Case 2. (a) Contrast-enhanced computed tomography image. T1-weighted axial (b) and T2-weighted sagittal (c) magnetic resonance images. Computed tomography and magnetic resonance images show a tumor with a clear margin and intralesional adipose tissue in the left lower neck. (d) Gross findings of the tumor. The tumor was encapsulated and had a smooth external surface.

A 24-year-old woman had been aware of a lower left neck swelling for several days. She underwent an open biopsy of the left neck by a previous physician. The histopathological examination suggested SS. The previous physician planned to perform surgery after chemotherapy or chemoradiotherapy. The patient was referred to our department to obtain more detailed examinations before surgery. On physical examination, a soft mass was palpated in the lower left neck. CT scans (Figure 3(a)) revealed a 42 × 35 × 48 mm well-circumscribed mass with nonhomogeneous enhancement between the sternocleidomastoid muscle and internal jugular vein. The mass had low signal intensity similar to muscle on T1WI (Figure 3(b)) and higher intensity than muscle on T2WI (Figure 3(c)). Densities and intensities resembling those of intralesional and marginal fat were observed. On FDG-PET/CT, there was no accumulation in the tumor. Upon histopathological examination of the open biopsy performed by the previous physician, the tumor consisted of comparatively even spindle cells and adipose-like cells. According to the immunohistochemical examination, the spindle cells were CK AE1/3 (+), *α*-SMA (+), S100 (−), desmin (−), Bcl2 (+), and TdT (−). The tumor was suspected of being SS. We used the Vysis SS18 Break-Apart FISH Probe Kit (Abbott Laboratories, Abbott Park, Illinois, USA). The gene was not disrupted in the sample, and SS was excluded from the differential diagnosis.

On the basis of the findings in Case 1, we suspected EHT, but a definitive diagnosis could not be obtained from the biopsy. Tumor resection from the left neck was performed under general anesthesia. The scar created by the previous open biopsy was also resected. Part of the sternocleidomastoid muscle between the skin scar and the tumor was also resected. The tumor (Figure 3(d)) was encapsulated with a smooth surface, and there was no adhesion to the surroundings. The surrounding organs were preserved, aside from the partial resection of the sternocleidomastoid muscle between the tumor and the scar, which was performed because the possibility of malignancy could not be excluded.

**Figure 4:**
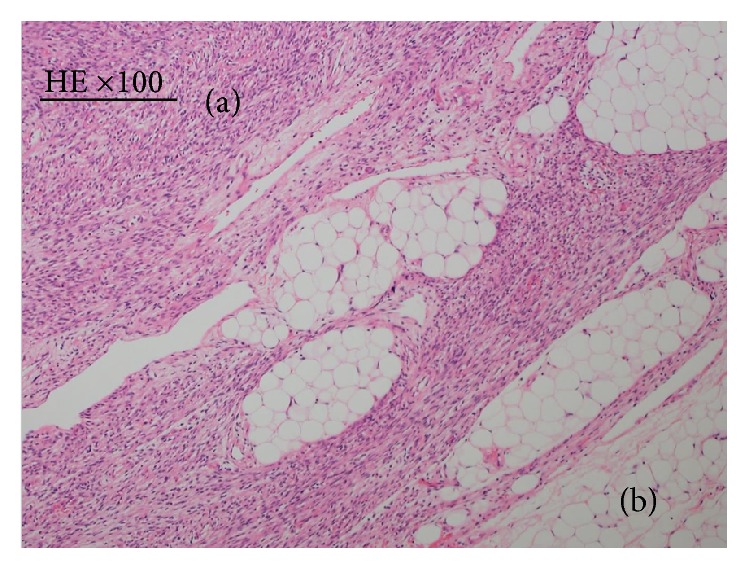
Hematoxylin and eosin (HE) staining findings in Case 2 (×100). (a) Plump to thin spindle cells with a low degree of cellular atypia and low mitotic activity can be observed. (b) Adipose cells can be observed within the tumor.

On intraoperative frozen section examination, spindle cells with no pleomorphism and adipose tissue were seen inside the lesion. Necrosis and mitotic figures were absent. These findings were compatible with a diagnosis of EHT. On histopathological examination of the resected tumor, the tumor consisted of plump to thin spindle cells growing in a bundle shape (Figure 4(a)) and adipose cells (Figure 4(b)). Based on immunohistochemical examination and fusion gene examination, we diagnosed the tumor as EHT. The patient has had no recurrence for 29 months after surgery. The patient provided written informed consent for all medical procedures and for the publication of this case report.

## 3. Discussion

EHT is a disease that has characteristics of both hamartomas and tumors. It was first described in 1982 by Smith and McClure [3] and Rosai et al. [4]. Chan and Rosal [5] defined EHT as a benign “tumor of the neck showing thymic or related branchial pouch differentiation.” There was no evidence that this tumor had any relationship with the thymus. Fetsch et al. [6] proposed that “branchial anlage mixed tumor” might be a better name for this disease.

EHT has a markedly higher incidence in men, with a male-to-female ratio of >10 to 1. The tumor most commonly affects adults, with a median age at diagnosis of 42.5 years. It occurs exclusively in the superficial or deep soft tissues of the supraclavicular, suprasternal, or presternal regions. Tumor development is slow, and the clinical course is long. Recurrence after complete surgical excision or metastasis is extremely rare, and most patients experience a benign clinical course [1].

Histopathologically, EHT consists of spindle cells, epithelial cells, and mature adipose cells. Immunohistochemically, both spindle cells and epithelial cells are CK AE1/3 (+), and the spindle cells are *α*-SMA (+). EHT lacks any population of immature T-cells, and TdT is negative in this tumor. In the histopathological differential diagnosis, SS is the most important tumor to exclude [1, 7]. Although the spindle cell arrays are similar in EHT and SS, SS has larger spindle cells and cellular atypia. In our cases, differentiating between SS and EHT was difficult, and the elimination of SS required fusion gene analysis using PCR or FISH. SS is characterized by the t(X;18)(p11;q11) translocation. This translocation or complex variants are present in more than 95% of all cases. Through this translocation, the SS18 gene (also known as SYT) localized on chromosome 18 and the SSX genes (SSX1, SSX2, or SSX4) localized on the X chromosome become fused, and a SYT-SSX chimeric gene is created. PCR and Break-Apart FISH for assessing fusion transcript expression have been employed widely for accurate diagnoses of SS [2]. We used frozen samples to test for SYT-SSX fusion gene expression via PCR in Case 1. We acquired the Vysis SS18 Break-Apart FISH Probe Kit between the times at which we encountered Case 1 and Case 2. Therefore, we were able to analyze a paraffin-embedded section and perform SS18 Break-Apart FISH for Case 2.

EHT has no specific radiological characteristics. In our cases, however, CT and MRI images showed well-marginated masses in the lower anterior neck with scattered fat density or intensity. Iida et al. [8] also reported the radiological differential diagnosis of fat-containing tumors. Mature adipose cells are pathological components of EHT. Therefore, we concluded that well-circumscribed masses with fat in the lower anterior neck were specific imaging features of EHT.

The treatment for EHT is usually simple resection [7]. Although EHT is a disease that should be differentiated as a lower anterior neck tumor, it may be misdiagnosed as a malignant tumor, such as a sarcoma, because it is so rare. If EHT is not properly diagnosed, it leads to potentially excessive treatment. On the other hand, if a malignant tumor cannot be excluded, we must avoid giving the patient too little treatment. Since a definitive diagnosis of EHT before surgery was impossible in Case 1, the resection included some of the surrounding organs. We strongly suspected EHT before surgery in Case 2 and, therefore, we were able to preserve the surrounding organs, except for the sternocleidomastoid muscle.

The ideal strategy for EHT is definitive diagnosis before surgery and simple resection to avoid excessive surgery. Regarding imaging findings, both tumor location (supraclavicular, suprasternal, or presternal region) and the presence of adipose inside the tumor are important. Regarding histopathological findings, spindle cells, epithelial cells, and mature adipose cells within the tumor are important, as is the exclusion of SS by fusion gene analysis using PCR or FISH.

In conclusion, EHT should be included in the differential diagnosis of lower anterior neck tumors. Fusion gene assessment using PCR or FISH is useful for differentiating between EHT and SS.
